# 4-[(2-Hy­droxy-5-nitro­benzyl­idene)amino]­benzene­sulfonamide

**DOI:** 10.1107/S1600536810036949

**Published:** 2010-09-25

**Authors:** Yi-Han Tan, Siang Guan Teoh, Wan-Sin Loh, Hoong-Kun Fun

**Affiliations:** a School of Chemical Sciences, Universiti Sains Malaysia, 11800 USM, Penang, Malaysia; b X-ray Crystallography Unit, School of Physics, Universiti Sains Malaysia, 11800 USM, Penang, Malaysia

## Abstract

The title Schiff base compound, C_13_H_11_N_3_O_5_S, exists in an *E* configuration with respect to the C=N double bond. The benzene rings are almost coplanar, making a dihedral angle of 2.82 (6). The sulfonamide group is twisted away from the attached phenyl ring with an N—S—C—C torsion angle of 64.84 (11)°. An intra­molecular O—H⋯N hydrogen bond stabilizes the mol­ecule, generating an *S*(6) ring motif. In the crystal, inter­molecular N—H⋯O and C—H⋯O hydrogen bonds link the mol­ecules into a three-dimensional network.

## Related literature

For background and the biological activity of sulfonamide and its derivatives, see: Kremer *et al.* (2006 ▶); Chumakov *et al.* (2006 ▶); Mohamed & Sharaby (2007 ▶); Wang *et al.* (2010 ▶); Sharaby (2007 ▶); Aziz-ur-Rehman *et al.* (2010 ▶); Subashini *et al.* (2009 ▶); Loughrey *et al.* (2009 ▶). For a related structure, see: Fun *et al.* (2010 ▶). For bond-length data, see: Allen *et al.* (1987 ▶). For hydrogen-bond motifs, see: Bernstein *et al.* (1995 ▶). For the stability of the temperature controller used in the data collection, see: Cosier & Glazer (1986 ▶).
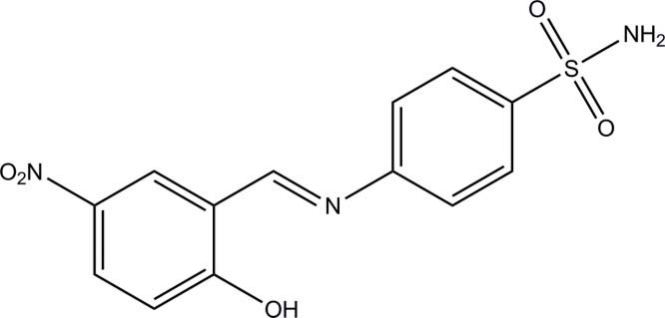


## Experimental

### 

#### Crystal data

C_13_H_11_N_3_O_5_S
*M* *_r_* = 321.31
Monoclinic, 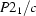

*a* = 6.7698 (1) Å
*b* = 26.4754 (3) Å
*c* = 9.9683 (1) Å
β = 131.544 (1)°
*V* = 1337.21 (3) Å^3^
*Z* = 4
Mo *K*α radiation
μ = 0.27 mm^−1^
*T* = 100 K
0.41 × 0.27 × 0.06 mm

#### Data collection

Bruker SMART APEXII CCD area-detector diffractometer
Absorption correction: multi-scan (*SADABS*; Bruker, 2009 ▶) *T*_min_ = 0.896, *T*_max_ = 0.985
29377 measured reflections
4825 independent reflections
4104 reflections with *I* > 2σ(*I*)
*R*_int_ = 0.031

#### Refinement

*R*[*F* ^2^ > 2σ(*F* ^2^)] = 0.039
*wR*(*F* ^2^) = 0.110
*S* = 1.06
4825 reflections
211 parameters
H atoms treated by a mixture of independent and constrained refinement
Δρ_max_ = 0.60 e Å^−3^
Δρ_min_ = −0.43 e Å^−3^

### 

Data collection: *APEX2* (Bruker, 2009 ▶); cell refinement: *SAINT* (Bruker, 2009 ▶); data reduction: *SAINT*; program(s) used to solve structure: *SHELXTL* (Sheldrick, 2008 ▶); program(s) used to refine structure: *SHELXTL*; molecular graphics: *SHELXTL*; software used to prepare material for publication: *SHELXTL* and *PLATON* (Spek, 2009 ▶).

## Supplementary Material

Crystal structure: contains datablocks global, I. DOI: 10.1107/S1600536810036949/sj5036sup1.cif

Structure factors: contains datablocks I. DOI: 10.1107/S1600536810036949/sj5036Isup2.hkl

Additional supplementary materials:  crystallographic information; 3D view; checkCIF report

## Figures and Tables

**Table 1:** Hydrogen-bond geometry (Å, °)

*D*—H⋯*A*	*D*—H	H⋯*A*	*D*⋯*A*	*D*—H⋯*A*
N2—H2*N*2⋯O5^i^	0.82 (3)	2.07 (3)	2.891 (2)	172 (3)
N2—H1*N*2⋯O2^ii^	0.84 (3)	2.58 (2)	3.1378 (15)	125 (2)
N2—H1*N*2⋯O4^iii^	0.84 (3)	2.11 (3)	2.883 (2)	153 (2)
O1—H1*O*1⋯N1	0.96 (3)	1.67 (3)	2.5626 (15)	154 (4)
C5—H5*A*⋯O3^iv^	0.93	2.55	3.3350 (17)	143
C7—H7*A*⋯O3^iv^	0.93	2.36	3.187 (2)	148
C10—H10*A*⋯O1^v^	0.93	2.58	3.1861 (19)	123

Symmetry codes: (i) 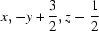; (ii) 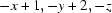; (iii) 
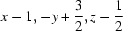
; (iv) 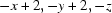; (v) 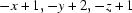.

**Fig. 1.**
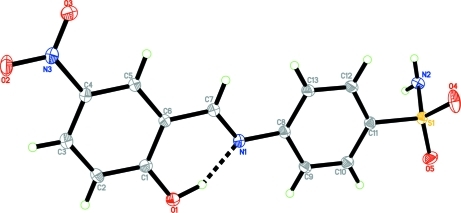
The molecular structure of the title compound, showing 50% probability displacement ellipsoids and the atom-numbering scheme. The dashed line indicates the intramolecular hydrogen bond.

**Fig. 2.**
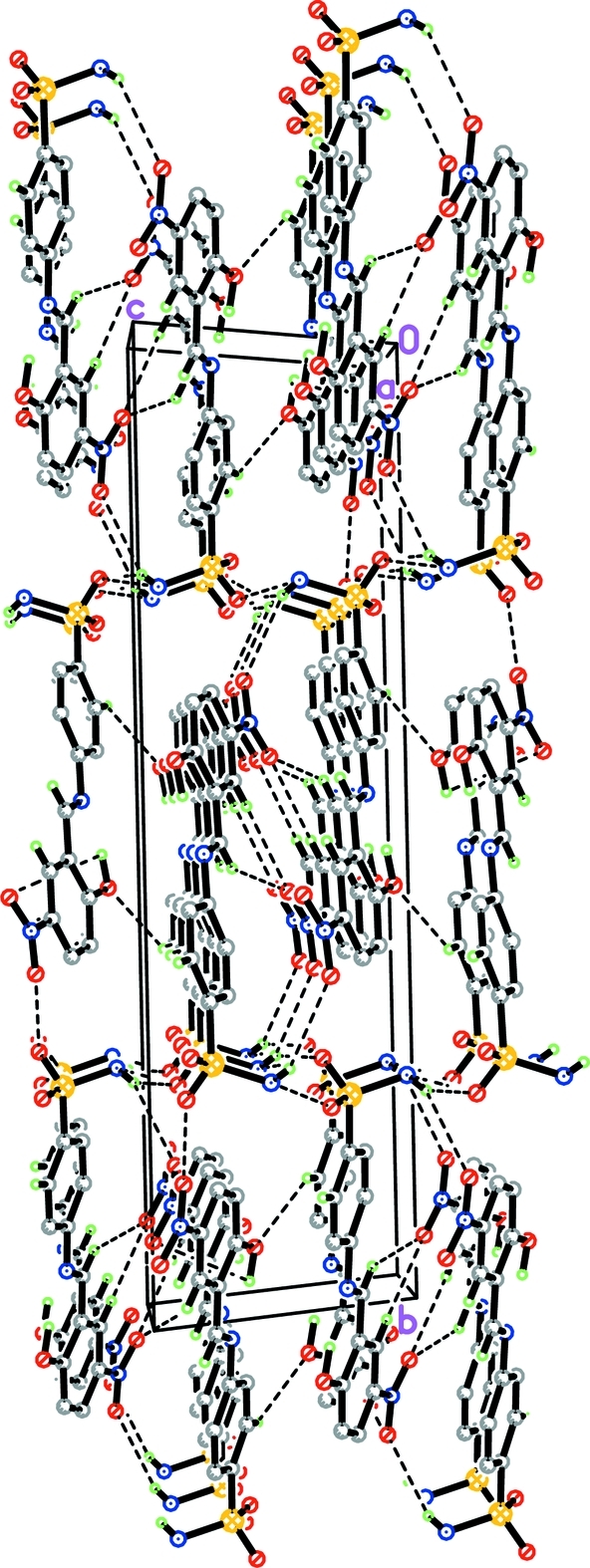
The crystal packing of the title compound, viewed along the a axis, showing the three-dimensional network. Intermolecular interactions are shown as dashed lines. H atoms not involved in the intermolecular interactions (dashed lines) have been omitted for clarity.

## supplementary crystallographic information

### Comment

Sulfanilamide which is also known as sulfonamide and its derivatives are extensively used in medicine as they possess a wide range of medicinal, pharmacological and antimicrobial properties (Kremer *et al.*, 2006, Chumakov *et al.*, 2006). Sulfanilamide was the first drug that had been found to be used as a preventive and therapeutic agent towards a variety of diseases or bacterial infections in human biological systems. Schiff bases derived from sulfanilamide exhibit various pharmacological activities including antibacterial, antifungal, antiviral, antimicrobial, anticonvulsant, antitumor, antiulcer, anti-neoplastic, and anti-inflammatory properties as well as acting as enzymatic inhibitors (Wang *et al.*, 2010, Sharaby, 2007, Kremer *et al.*, 2006, Chumakov *et al.*, 2006, Aziz-ur-Rehman *et al.*, 2010, Subashini *et al.*, 2009, Loughrey *et al.*, 2009). The biological activity could be increased by the complexation with metal ions (Mohamed & Sharaby, 2007; Kremer *et al.*, 2006).

The title Schiff base compound (Fig. 1) exists in an *E* configuration with respect to the C7═N1 double bond. The phenyl rings (C1–C6 & C8–C13) are almost coplanar with each other with a dihedral angle of 2.82 (6)°. The sulfonamide group (S1/O3/O4/N2) is twisted away from the attached phenyl ring with the torsion angle between N2–S1–C11–C12 being 64.84 (11)°. An intramolecular O1—H1O1···N1 hydrogen bond stabilized the molecule, generating an *S*(6) ring motif (Bernstein *et al.*, 1995). Bond lengths (Allen *et al.*, 1987) and angles are within the normal ranges and are comparable to the related structure (Fun *et al.*, 2010).

In the crystal packing (Fig. 2), intermolecular N2—H2N2···O5, N2—H1N2···O2, N2—H1N2···O4, C5—H5A···O3, C7—H7A···O3 and C10—H10A···O1 hydrogen bonds (Table 1) link the molecules into three-dimensional network.

### Experimental

The title compound was synthesized by the general method of condensation of sulfanilamide with 2-hydroxy-5-nitrobenzaldehyde. To a methanolic solution (10 ml) of sulfanilamide (0.3444 g, 2 mmol), sodium acetate (0.1641 g, 2 mmol) was added. The resulting solution was heated to reflux on a water bath. A methanolic solution (10 ml) of 2-hydroxy-5-nitrobenzaldehyde (0.3342 g, 2 mmol) was then added dropwise to the solution. The solution was left under reflux (65–70°C) for 1 h. After half an hour, yellowish orange precipitate started to form during refluxing. The resulting mixture was filtered and the filtrate was left to evaporate slowly at room temperature. Orange crystals of the title compound suitable for X-ray diffraction were obtained after 1 week.

### Refinement

N– and O– bound H atoms were located from difference Fourier map and refined freely with the  N–H  bond lengths being 0.83 (2) to 0.84 (2) Å and the O–H distance 0.96 (3) Å. The remaining H atoms were positioned geometrically and refined using a riding model, with *U*_iso_(H) = 1.2 *U*_eq_(C) [C–H = 0.93 Å].

### Crystal data

**Table:**

C_13_H_11_N_3_O_5_S	*F*(000) = 664
*M**_r_* = 321.31	*D*_x_ = 1.596 Mg m^−^^3^
Monoclinic, *P*2_1_/*c*	Mo *K*α radiation, λ = 0.71073 Å
Hall symbol: -P 2ybc	Cell parameters from 9950 reflections
*a* = 6.7698 (1) Å	θ = 2.8–35.5°
*b* = 26.4754 (3) Å	µ = 0.27 mm^−^^1^
*c* = 9.9683 (1) Å	*T* = 100 K
β = 131.544 (1)°	Plate, orange
*V* = 1337.21 (3) Å^3^	0.41 × 0.27 × 0.06 mm
*Z* = 4	

### Data collection

**Table:**

Bruker SMART APEXII CCD area-detector diffractometer	4825 independent reflections
Radiation source: fine-focus sealed tube	4104 reflections with *I* > 2σ(*I*)
graphite	*R*_int_ = 0.031
φ and ω scans	θ_max_ = 32.5°, θ_min_ = 2.8°
Absorption correction: multi-scan (*SADABS*; Bruker, 2009)	*h* = −10→10
*T*_min_ = 0.896, *T*_max_ = 0.985	*k* = −40→36
29377 measured reflections	*l* = −15→15

### Refinement

**Table:**

Refinement on *F*^2^	Primary atom site location: structure-invariant direct methods
Least-squares matrix: full	Secondary atom site location: difference Fourier map
*R*[*F*^2^ > 2σ(*F*^2^)] = 0.039	Hydrogen site location: inferred from neighbouring sites
*wR*(*F*^2^) = 0.110	H atoms treated by a mixture of independent and constrained refinement
*S* = 1.06	*w* = 1/[σ^2^(*F*_o_^2^) + (0.0528*P*)^2^ + 0.7191*P*] where *P* = (*F*_o_^2^ + 2*F*_c_^2^)/3
4825 reflections	(Δ/σ)_max_ = 0.002
211 parameters	Δρ_max_ = 0.60 e Å^−^^3^
0 restraints	Δρ_min_ = −0.43 e Å^−^^3^

### Special details

**Table:**

Experimental. The crystal was placed in the cold stream of an Oxford Cyrosystems Cobra open-flow nitrogen cryostat (Cosier & Glazer, 1986) operating at 100.0 (1) K.
Geometry. All e.s.d.'s (except the e.s.d. in the dihedral angle between two l.s. planes) are estimated using the full covariance matrix. The cell e.s.d.'s are taken into account individually in the estimation of e.s.d.'s in distances, angles and torsion angles; correlations between e.s.d.'s in cell parameters are only used when they are defined by crystal symmetry. An approximate (isotropic) treatment of cell e.s.d.'s is used for estimating e.s.d.'s involving l.s. planes.
Refinement. Refinement of *F*^2^ against ALL reflections. The weighted *R*-factor *wR* and goodness of fit *S* are based on *F*^2^, conventional *R*-factors *R* are based on *F*, with *F* set to zero for negative *F*^2^. The threshold expression of *F*^2^ > σ(*F*^2^) is used only for calculating *R*-factors(gt) *etc*. and is not relevant to the choice of reflections for refinement. *R*-factors based on *F*^2^ are statistically about twice as large as those based on *F*, and *R*- factors based on ALL data will be even larger.

### Fractional atomic coordinates and isotropic or equivalent isotropic displacement parameters (Å^2^)

**Table:**

	*x*	*y*	*z*	*U*_iso_*/*U*_eq_	
S1	0.25811 (6)	0.770137 (11)	0.29838 (4)	0.01433 (8)	
O1	0.69814 (19)	1.06412 (4)	0.38905 (13)	0.01833 (18)	
O2	1.2322 (2)	1.14670 (4)	0.11034 (15)	0.0251 (2)	
O3	1.1687 (2)	1.06896 (4)	0.02207 (13)	0.0217 (2)	
O4	0.46897 (19)	0.73446 (4)	0.38253 (15)	0.0264 (2)	
O5	0.1385 (2)	0.77870 (4)	0.37367 (14)	0.0243 (2)	
N1	0.6474 (2)	0.97162 (4)	0.29501 (13)	0.01443 (19)	
N2	0.0283 (2)	0.75128 (4)	0.09717 (14)	0.01538 (19)	
N3	1.1534 (2)	1.10368 (4)	0.09747 (14)	0.0166 (2)	
C1	0.8033 (2)	1.07306 (5)	0.31518 (15)	0.0146 (2)	
C2	0.8745 (2)	1.12251 (5)	0.31389 (16)	0.0169 (2)	
H2A	0.8429	1.1487	0.3599	0.020*	
C3	0.9919 (2)	1.13262 (5)	0.24433 (16)	0.0167 (2)	
H3A	1.0412	1.1654	0.2442	0.020*	
C4	1.0352 (2)	1.09290 (5)	0.17442 (15)	0.0142 (2)	
C5	0.9623 (2)	1.04378 (5)	0.17048 (15)	0.0146 (2)	
H5A	0.9913	1.0181	0.1215	0.018*	
C6	0.8442 (2)	1.03309 (5)	0.24119 (15)	0.0139 (2)	
C7	0.7632 (2)	0.98178 (5)	0.23515 (16)	0.0158 (2)	
H7A	0.7949	0.9561	0.1879	0.019*	
C8	0.5618 (2)	0.92230 (5)	0.29004 (15)	0.0135 (2)	
C9	0.4354 (2)	0.91795 (5)	0.35687 (16)	0.0152 (2)	
H9A	0.4130	0.9465	0.4000	0.018*	
C10	0.3425 (2)	0.87159 (5)	0.35986 (16)	0.0158 (2)	
H10A	0.2583	0.8689	0.4045	0.019*	
C11	0.3773 (2)	0.82917 (5)	0.29516 (15)	0.0139 (2)	
C12	0.5046 (2)	0.83273 (5)	0.22825 (16)	0.0163 (2)	
H12A	0.5280	0.8040	0.1862	0.020*	
C13	0.5958 (2)	0.87914 (5)	0.22483 (16)	0.0164 (2)	
H13A	0.6792	0.8818	0.1796	0.020*	
H2N2	0.074 (4)	0.7439 (7)	0.041 (3)	0.028 (5)*	
H1N2	−0.119 (4)	0.7655 (8)	0.038 (3)	0.030 (5)*	
H1O1	0.655 (5)	1.0290 (11)	0.368 (4)	0.068 (8)*	

### Atomic displacement parameters (Å^2^)

**Table:**

	*U*^11^	*U*^22^	*U*^33^	*U*^12^	*U*^13^	*U*^23^
S1	0.01492 (14)	0.01229 (14)	0.01464 (13)	−0.00158 (10)	0.00933 (11)	0.00082 (9)
O1	0.0243 (5)	0.0160 (5)	0.0229 (4)	0.0000 (4)	0.0191 (4)	−0.0007 (3)
O2	0.0303 (5)	0.0155 (5)	0.0373 (5)	−0.0009 (4)	0.0257 (5)	0.0052 (4)
O3	0.0270 (5)	0.0219 (5)	0.0227 (4)	−0.0038 (4)	0.0192 (4)	−0.0036 (4)
O4	0.0152 (4)	0.0161 (5)	0.0320 (5)	0.0019 (4)	0.0090 (4)	0.0079 (4)
O5	0.0372 (6)	0.0225 (5)	0.0267 (5)	−0.0090 (4)	0.0268 (5)	−0.0048 (4)
N1	0.0162 (4)	0.0123 (5)	0.0157 (4)	−0.0014 (4)	0.0110 (4)	−0.0003 (3)
N2	0.0137 (4)	0.0156 (5)	0.0161 (4)	−0.0005 (4)	0.0096 (4)	−0.0019 (4)
N3	0.0167 (5)	0.0155 (5)	0.0175 (4)	0.0006 (4)	0.0112 (4)	0.0032 (4)
C1	0.0147 (5)	0.0149 (5)	0.0142 (4)	0.0010 (4)	0.0096 (4)	0.0005 (4)
C2	0.0200 (5)	0.0117 (5)	0.0197 (5)	0.0014 (4)	0.0134 (5)	−0.0002 (4)
C3	0.0170 (5)	0.0134 (5)	0.0181 (5)	0.0001 (4)	0.0110 (5)	0.0010 (4)
C4	0.0148 (5)	0.0132 (5)	0.0146 (5)	−0.0003 (4)	0.0098 (4)	0.0011 (4)
C5	0.0179 (5)	0.0118 (5)	0.0154 (5)	−0.0005 (4)	0.0116 (4)	−0.0002 (4)
C6	0.0165 (5)	0.0118 (5)	0.0143 (4)	−0.0007 (4)	0.0105 (4)	−0.0006 (4)
C7	0.0195 (5)	0.0134 (5)	0.0171 (5)	−0.0009 (4)	0.0132 (5)	−0.0005 (4)
C8	0.0136 (5)	0.0122 (5)	0.0137 (4)	−0.0007 (4)	0.0087 (4)	−0.0002 (4)
C9	0.0183 (5)	0.0127 (5)	0.0185 (5)	−0.0005 (4)	0.0138 (5)	−0.0016 (4)
C10	0.0173 (5)	0.0158 (6)	0.0178 (5)	−0.0015 (4)	0.0132 (5)	−0.0012 (4)
C11	0.0143 (5)	0.0129 (5)	0.0139 (4)	−0.0017 (4)	0.0091 (4)	−0.0005 (4)
C12	0.0196 (5)	0.0138 (5)	0.0187 (5)	−0.0021 (4)	0.0140 (5)	−0.0030 (4)
C13	0.0202 (5)	0.0146 (6)	0.0199 (5)	−0.0020 (4)	0.0157 (5)	−0.0022 (4)

### Geometric parameters (Å, °)

**Table:**

S1—O4	1.4301 (10)	C3—C4	1.3958 (17)
S1—O5	1.4408 (10)	C3—H3A	0.9300
S1—N2	1.5980 (11)	C4—C5	1.3822 (17)
S1—C11	1.7689 (12)	C5—C6	1.4014 (15)
O1—C1	1.3417 (14)	C5—H5A	0.9300
O1—H1O1	0.96 (3)	C6—C7	1.4513 (17)
O2—N3	1.2278 (14)	C7—H7A	0.9300
O3—N3	1.2345 (14)	C8—C9	1.3951 (15)
N1—C7	1.2892 (15)	C8—C13	1.4068 (16)
N1—C8	1.4160 (16)	C9—C10	1.3881 (17)
N2—H2N2	0.83 (2)	C9—H9A	0.9300
N2—H1N2	0.84 (2)	C10—C11	1.3908 (17)
N3—C4	1.4576 (15)	C10—H10A	0.9300
C1—C2	1.3982 (17)	C11—C12	1.3999 (16)
C1—C6	1.4181 (16)	C12—C13	1.3855 (17)
C2—C3	1.3837 (17)	C12—H12A	0.9300
C2—H2A	0.9300	C13—H13A	0.9300
			
O4—S1—O5	119.05 (7)	C4—C5—H5A	120.4
O4—S1—N2	106.97 (6)	C6—C5—H5A	120.4
O5—S1—N2	106.71 (6)	C5—C6—C1	118.97 (11)
O4—S1—C11	107.71 (6)	C5—C6—C7	119.61 (10)
O5—S1—C11	106.92 (6)	C1—C6—C7	121.42 (10)
N2—S1—C11	109.23 (6)	N1—C7—C6	120.34 (11)
C1—O1—H1O1	104.2 (17)	N1—C7—H7A	119.8
C7—N1—C8	122.45 (11)	C6—C7—H7A	119.8
S1—N2—H2N2	115.9 (13)	C9—C8—C13	119.57 (11)
S1—N2—H1N2	117.2 (14)	C9—C8—N1	115.21 (10)
H2N2—N2—H1N2	115.7 (19)	C13—C8—N1	125.21 (10)
O2—N3—O3	123.25 (11)	C10—C9—C8	120.88 (11)
O2—N3—C4	118.61 (11)	C10—C9—H9A	119.6
O3—N3—C4	118.14 (10)	C8—C9—H9A	119.6
O1—C1—C2	118.81 (11)	C9—C10—C11	119.06 (10)
O1—C1—C6	120.75 (11)	C9—C10—H10A	120.5
C2—C1—C6	120.44 (11)	C11—C10—H10A	120.5
C3—C2—C1	120.11 (11)	C10—C11—C12	120.90 (11)
C3—C2—H2A	119.9	C10—C11—S1	119.05 (9)
C1—C2—H2A	119.9	C12—C11—S1	120.04 (9)
C2—C3—C4	119.01 (11)	C13—C12—C11	119.77 (11)
C2—C3—H3A	120.5	C13—C12—H12A	120.1
C4—C3—H3A	120.5	C11—C12—H12A	120.1
C5—C4—C3	122.27 (11)	C12—C13—C8	119.81 (10)
C5—C4—N3	118.65 (11)	C12—C13—H13A	120.1
C3—C4—N3	119.05 (11)	C8—C13—H13A	120.1
C4—C5—C6	119.17 (11)		
			
O1—C1—C2—C3	177.73 (11)	C1—C6—C7—N1	1.12 (18)
C6—C1—C2—C3	−1.66 (18)	C7—N1—C8—C9	−178.76 (11)
C1—C2—C3—C4	0.62 (18)	C7—N1—C8—C13	1.41 (19)
C2—C3—C4—C5	0.68 (19)	C13—C8—C9—C10	−0.02 (18)
C2—C3—C4—N3	178.64 (11)	N1—C8—C9—C10	−179.86 (11)
O2—N3—C4—C5	−175.78 (11)	C8—C9—C10—C11	0.06 (19)
O3—N3—C4—C5	4.36 (16)	C9—C10—C11—C12	0.21 (18)
O2—N3—C4—C3	6.19 (17)	C9—C10—C11—S1	−179.82 (9)
O3—N3—C4—C3	−173.67 (11)	O4—S1—C11—C10	−128.97 (10)
C3—C4—C5—C6	−0.92 (18)	O5—S1—C11—C10	0.07 (12)
N3—C4—C5—C6	−178.88 (10)	N2—S1—C11—C10	115.18 (10)
C4—C5—C6—C1	−0.13 (17)	O4—S1—C11—C12	51.00 (12)
C4—C5—C6—C7	179.03 (11)	O5—S1—C11—C12	−179.95 (10)
O1—C1—C6—C5	−177.97 (11)	N2—S1—C11—C12	−64.84 (11)
C2—C1—C6—C5	1.41 (18)	C10—C11—C12—C13	−0.51 (18)
O1—C1—C6—C7	2.88 (18)	S1—C11—C12—C13	179.51 (10)
C2—C1—C6—C7	−177.74 (11)	C11—C12—C13—C8	0.55 (19)
C8—N1—C7—C6	179.21 (11)	C9—C8—C13—C12	−0.29 (18)
C5—C6—C7—N1	−178.02 (11)	N1—C8—C13—C12	179.54 (11)

### Hydrogen-bond geometry (Å, °)

**Table:**

*D*—H···*A*	*D*—H	H···*A*	*D*···*A*	*D*—H···*A*
N2—H2N2···O5^i^	0.82 (3)	2.07 (3)	2.891 (2)	172 (3)
N2—H1N2···O2^ii^	0.84 (3)	2.58 (2)	3.1378 (15)	125 (2)
N2—H1N2···O4^iii^	0.84 (3)	2.11 (3)	2.883 (2)	153 (2)
O1—H1O1···N1	0.96 (3)	1.67 (3)	2.5626 (15)	154 (4)
C5—H5A···O3^iv^	0.93	2.55	3.3350 (17)	143
C7—H7A···O3^iv^	0.93	2.36	3.187 (2)	148
C10—H10A···O1^v^	0.93	2.58	3.1861 (19)	123

Symmetry codes: (i) *x*, −*y*+3/2, *z*−1/2; (ii) −*x*+1, −*y*+2, −*z*; (iii) *x*−1, −*y*+3/2, *z*−1/2; (iv) −*x*+2, −*y*+2, −*z*; (v) −*x*+1, −*y*+2, −*z*+1.

## References

[bb1] Allen, F. H., Kennard, O., Watson, D. G., Brammer, L., Orpen, A. G. & Taylor, R. (1987). *J. Chem. Soc. Perkin Trans. 2*, pp. S1–19.

[bb2] Aziz-ur-Rehman, Sajjad, M. A., Akkurt, M., Sharif, S., Abbasi, M. A. & Khan, I. U. (2010). *Acta Cryst.* E**66**, o1769. 10.1107/S1600536810023871 PMC3006884 21587983

[bb3] Bernstein, J., Davis, R. E., Shimoni, L. & Chang, N.-L. (1995). *Angew. Chem. Int. Ed. Engl.* **34**, 1555–1573.

[bb4] Bruker (2009). *APEX2*, *SAINT* and *SADABS* Bruker AXS Inc., Madison, Wisconsin, USA.

[bb5] Chumakov, Y. M., Tsapkov, V. I., Bocelli, G., Antonsyak, B. Y., Palomares-Sánches, S. A., Ortiz, R. S. & Gulya, A. P. (2006). *J. Struct. Chem.* **47**, 923–929.

[bb6] Cosier, J. & Glazer, A. M. (1986). *J. Appl. Cryst.* **19**, 105–107.

[bb7] Fun, H.-K., Goh, J. H., Chidan Kumar, C. S., Yathirajan, H. S. & Narayana, B. (2010). *Acta Cryst.* E**66**, o372–o373. 10.1107/S1600536810001121 PMC2979666 21579795

[bb8] Kremer, E., Facchin, G., Estévez, E., Alborés, P., Baran, E. J., Ellena, J. & Torre, M. H. (2006). *J. Inorg. Biochem.* **100**, 1167–1175. 10.1016/j.jinorgbio.2006.01.042 16584779

[bb9] Loughrey, B. T., Williams, M. L. & Healy, P. C. (2009). *Acta Cryst.* E**65**, o2087. 10.1107/S1600536809030256 PMC2970159 21577505

[bb10] Mohamed, G. G. & Sharaby, C. M. (2007). *Spectrochim. Acta Part A*, **66**, 949–958. 10.1016/j.saa.2006.04.033 16891151

[bb11] Sharaby, C. M. (2007). *Spectrochim. Acta Part A*, **66**, 1271–1278. 10.1016/j.saa.2006.05.030 16920390

[bb12] Sheldrick, G. M. (2008). *Acta Cryst.* A**64**, 112–122. 10.1107/S0108767307043930 18156677

[bb13] Spek, A. L. (2009). *Acta Cryst.* D**65**, 148–155. 10.1107/S090744490804362X PMC2631630 19171970

[bb14] Subashini, A., Hemamalini, M., Muthiah, P. T., Bocelli, G. & Cantoni, A. (2009). *J. Chem. Crystallogr.* **39**, 112–116.

[bb15] Wang, X. L., Wan, K. & Zhou, C. H. (2010). *Eur. J. Med. Chem.* **45**, 4631–4639. 10.1016/j.ejmech.2010.07.031 20708826

